# Supplementary material for: MCL1 modulates mTORC1 signaling to promote bioenergetics and tumorigenesis
Source: Nat Commun. 2025 Dec 1;16:10841. doi: 10.1038/s41467-025-66831-4 (PMC12673096; doi:10.1038/s41467-025-66831-4)
Supplement: Supplementary file 1 — Supplementary Information [file 41467_2025_66831_MOESM1_ESM.pdf]

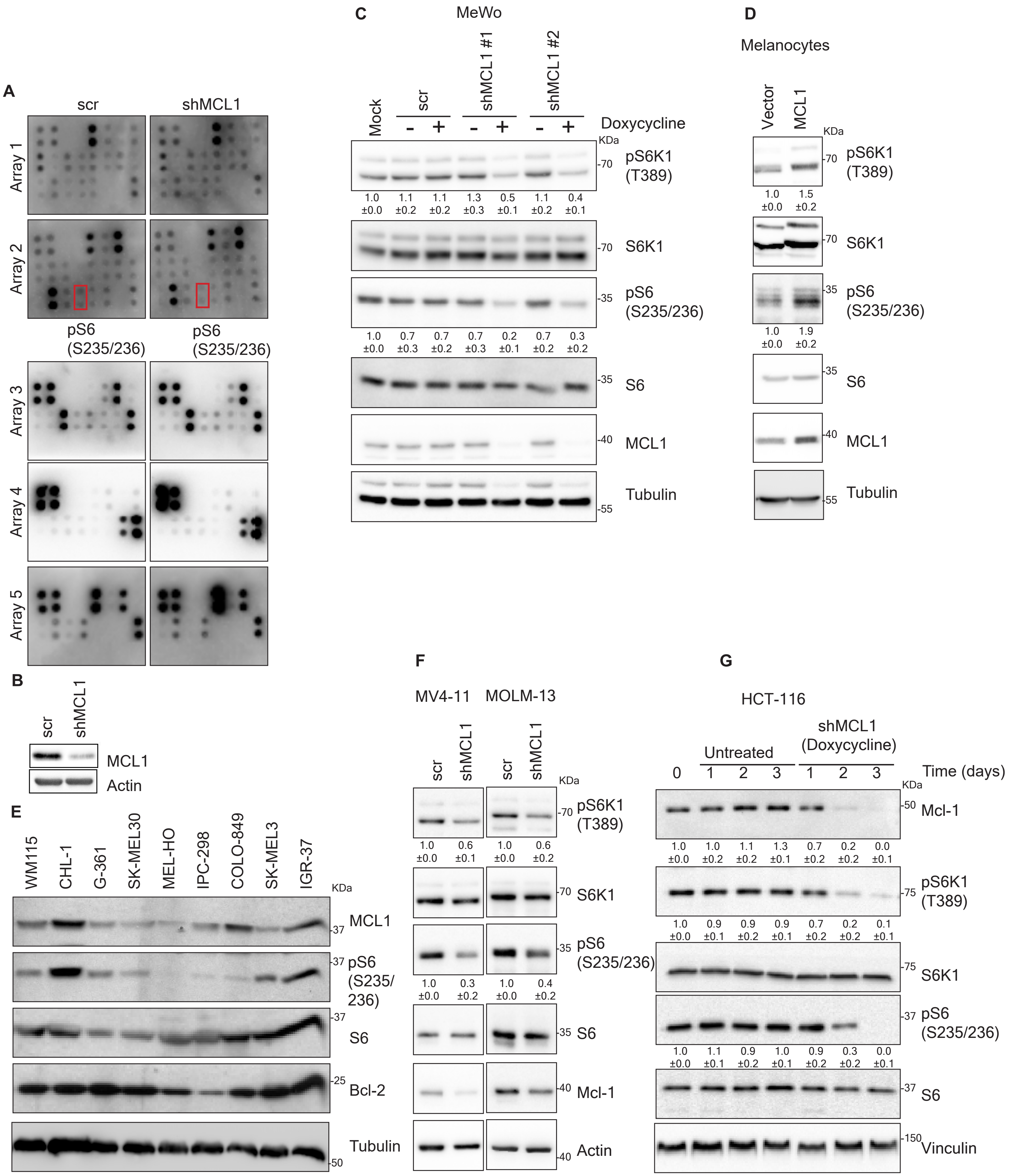
A
scr
shMCL1
Array 1
Array 2
pS6
(S235/236)
pS6
(S235/236)
Array 3
Array 4
Array 5
B
scr
shMCL1
MCL1
Actin
C
MeWo
Mock
scr
shMCL1 #1
shMCL1 #2
Doxycycline
- + - + - +
KDa
pS6K1
(T389)
1.0 ±0.0
1.1 ±0.2
1.1 ±0.2
1.3 ±0.3
0.5 ±0.1
1.1 ±0.2
0.4 ±0.1
S6K1
pS6
(S235/236)
1.0 ±0.0
0.7 ±0.3
0.7 ±0.2
0.7 ±0.3
0.2 ±0.1
0.7 ±0.2
0.3 ±0.2
S6
MCL1
Tubulin
-70
-70
-35
-35
-40
-55
D
Melanocytes
Vector
MCL1
KDa
pS6K1
(T389)
1.0 ±0.0
1.5 ±0.2
S6K1
pS6
(S235/236)
1.0 ±0.0
1.9 ±0.2
S6
MCL1
Tubulin
E
WM115
CHL-1
G-361
SK-MEL30
MEL-HO
IPC-298
COLO-849
SK-MEL3
IGR-37
KDa
MCL1
pS6
(S235/
236)
S6
Bcl-2
Tubulin
-37
-37
-37
-25
-50
F
MV4-11
MOLM-13
scr
shMCL1
scr
shMCL1
KDa
pS6K1
(T389)
1.0 ±0.0
0.6 ±0.1
1.0 ±0.0
0.6 ±0.2
S6K1
pS6
(S235/236)
1.0 ±0.0
0.3 ±0.2
1.0 ±0.0
0.4 ±0.2
S6
Mcl-1
Actin
G
HCT-116
Untreated
shMCL1
(Doxycycline)
0
1 2 3
1 2 3
Time (days)
KDa
Mcl-1
1.0 ±0.0
1.0 ±0.2
1.1 ±0.2
1.3 ±0.1
0.7 ±0.2
0.2 ±0.2
0.0 ±0.1
pS6K1
(T389)
1.0 ±0.0
0.9 ±0.1
0.9 ±0.2
0.9 ±0.1
0.7 ±0.2
0.2 ±0.2
0.1 ±0.1
S6K1
pS6
(S235/236)
1.0 ±0.0
1.1 ±0.1
0.9 ±0.2
1.0 ±0.1
0.9 ±0.2
0.3 ±0.2
0.0 ±0.1
S6
Vinculin
-50
-75
-75
-37
-37
-150

Supplementary Fig. 1. MCL1 knockdown inhibits mTORC1 signaling in several cell models.

(A) Phospho-kinase array analysis of lysate derived from CHL-1 melanoma cells transduced either with scrambled shRNA or shRNA against MCL1 for 72 hours, identifying modulation of mTORC1 target S6. Arrays 1-5 denotes the arrays included in the commercially available kit (RayBio® C-Series Human Phosphorylation Multi-Pathway Profiling Array C55). **(B)** Immunoblotting analysis of lysates of CHL-1 cells used in (A), validating the knockdown of MCL1. **(C)** Immunoblotting analysis of lysates derived from MeWo cells transduced with either scrambled shRNA or doxycycline-inducible shRNAs against MCL1 and treated with or without 500 ng/ml doxycycline for 72 hours. The samples derived from the same experiment but different gels for pS6K1, pS6, MCL1 and another for S6K1, S6 were processed in parallel. **(D)** Immunoblotting analysis of lysates of melanocytes expressing empty vector or MCL1 constructs. The samples derived from the same experiment but different gels for pS6K1, MCL1, another for pS6, another for S6K1 and another for S6 were processed in parallel. **(E)** Immunoblotting analysis of cell lysates derived from melanoma cells grown to comparable confluences. **(F)** Immunoblotting analysis of lysates derived from MV4-11 and MOLM-13 AML cells transduced with either scrambled shRNA or shRNA against MCL1 for 72 hours. The samples derived from the same experiment but different gels for pS6K1, MCL1, another for pS6, another for S6K1 and another for S6 were processed in parallel. **(G)** Immunoblotting analysis of lysates derived from HCT116 cells transduced with doxycycline-inducible shRNA against MCL1 and treated with or without 500 ng/ml doxycycline for the indicated time points. The samples derived from the same experiment but different gels for pS6K1, MCL1, another for pS6, another for S6K1 and another for S6 were processed in parallel.

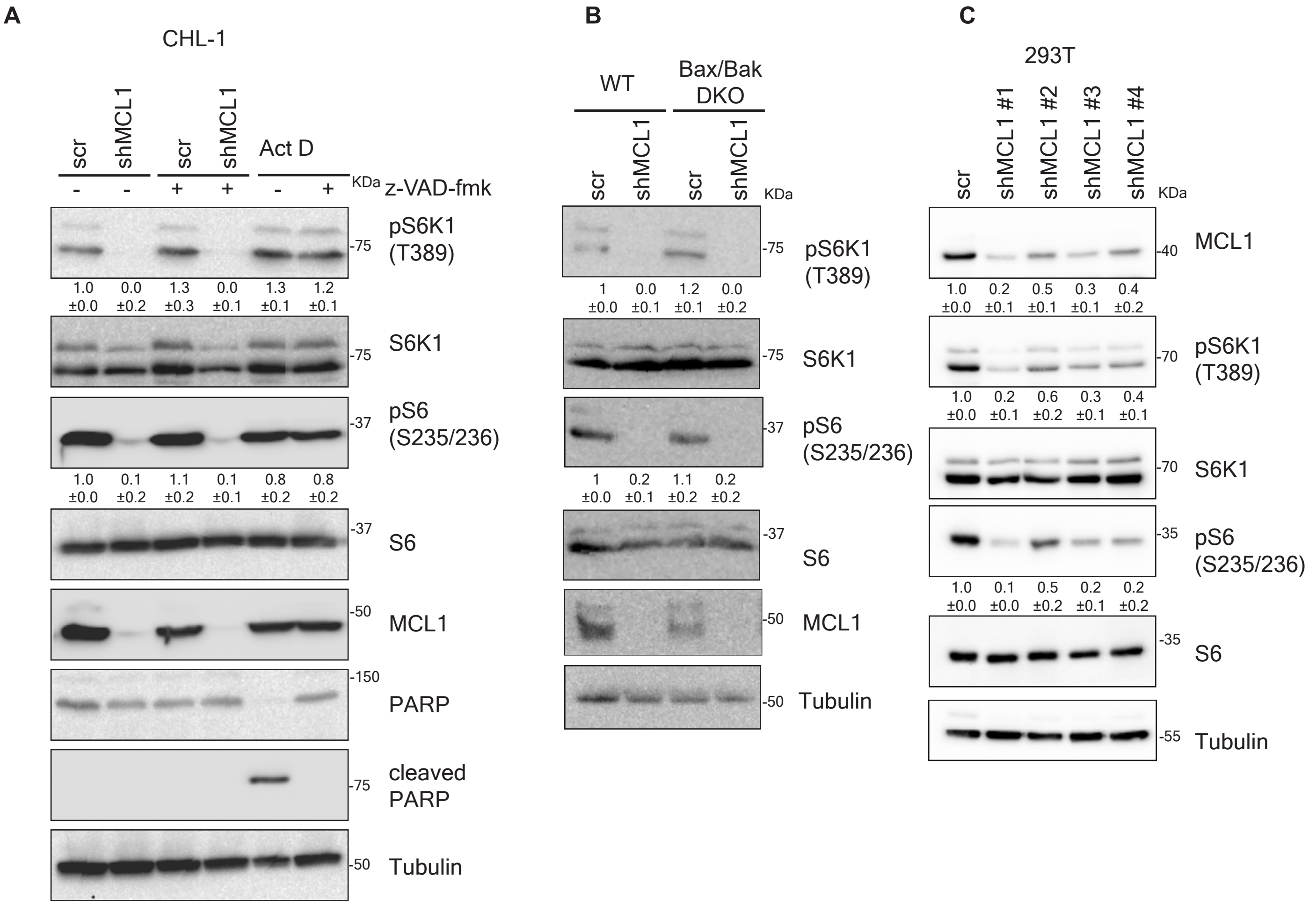
A
CHL-1
scr
shMCL1
scr
shMCL1
Act D
- - + + - +
KDa
z-VAD-fmk
pS6K1 (T389)
-75
1.0 ±0.0
0.0 ±0.2
1.3 ±0.3
0.0 ±0.1
1.3 ±0.1
1.2 ±0.1
S6K1
-75
pS6 (S235/236)
-37
1.0 ±0.0
0.1 ±0.2
1.1 ±0.2
0.1 ±0.1
0.8 ±0.2
0.8 ±0.2
S6
-37
MCL1
-50
PARP
-150
cleaved PARP
-75
Tubulin
-50
B
WT
Bax/Bak DKO
scr
shMCL1
scr
shMCL1
KDa
pS6K1 (T389)
-75
1 ±0.0
0.0 ±0.1
1.2 ±0.1
0.0 ±0.2
S6K1
-75
pS6 (S235/236)
-37
1 ±0.0
0.2 ±0.1
1.1 ±0.2
0.2 ±0.2
S6
-37
MCL1
-50
Tubulin
-50
C
293T
scr
shMCL1 #1
shMCL1 #2
shMCL1 #3
shMCL1 #4
KDa
MCL1
-40
1.0 ±0.0
0.2 ±0.1
0.5 ±0.1
0.3 ±0.1
0.4 ±0.2
pS6K1 (T389)
-70
1.0 ±0.0
0.2 ±0.1
0.6 ±0.2
0.3 ±0.1
0.4 ±0.1
S6K1
-70
pS6 (S235/236)
-35
1.0 ±0.0
0.1 ±0.0
0.5 ±0.2
0.2 ±0.1
0.2 ±0.2
S6
-35
Tubulin
-55

Supplementary Fig. 2. mTORC1 inhibition upon MCL1 knockdown is not associated with apoptosis

(A) Immunoblotting analysis of lysates prepared from CHL-1 cells transduced with either scrambled shRNA or shRNA against MCL1 for 72 hours showing modulation of mTORC1 in MCL1-depleted cells independent of apoptosis. Lysates from CHL-1 cells treated with Actinomycin D (Act D; 2 μM) for 24 hours were used as a positive control for the induction of apoptosis as indicated by cleavage of PARP protein. Treatment with pan-caspase inhibitor zVAD-fmk (20μM) for the last 16 hours was used as a control for inhibition of apoptosis. The samples derived from the same experiment but different gels for pS6K1, S6K1, another for pS6, another for S6, another for PARP and another for cleaved PARP were processed in parallel. **(B)** Immunoblotting analysis of lysates derived from wild-type (WT) or Bax/Bak double knockout (Bax/Bak DKO) MEFs transduced with either scrambled shRNA or shRNA against MCL1 for 72 hours. The samples derived from the same experiment but different gels for pS6K1, another for pS6, S6K1, another for S6 and another for MCL1were processed in parallel. **(C)** Immunoblotting analysis of lysate derived from HEK-293T cells transduced with different shRNA constructs against MCL1 with varying knockdown efficiency for 72 hours demonstrating a close correlation between the achievable levels of MCL1 knockdown and the magnitude of mTORC1 inhibition. The samples derived from the same experiment but different gels for pS6K1, MCL1, another for S6K1, pS6 and another for S6 were processed in parallel

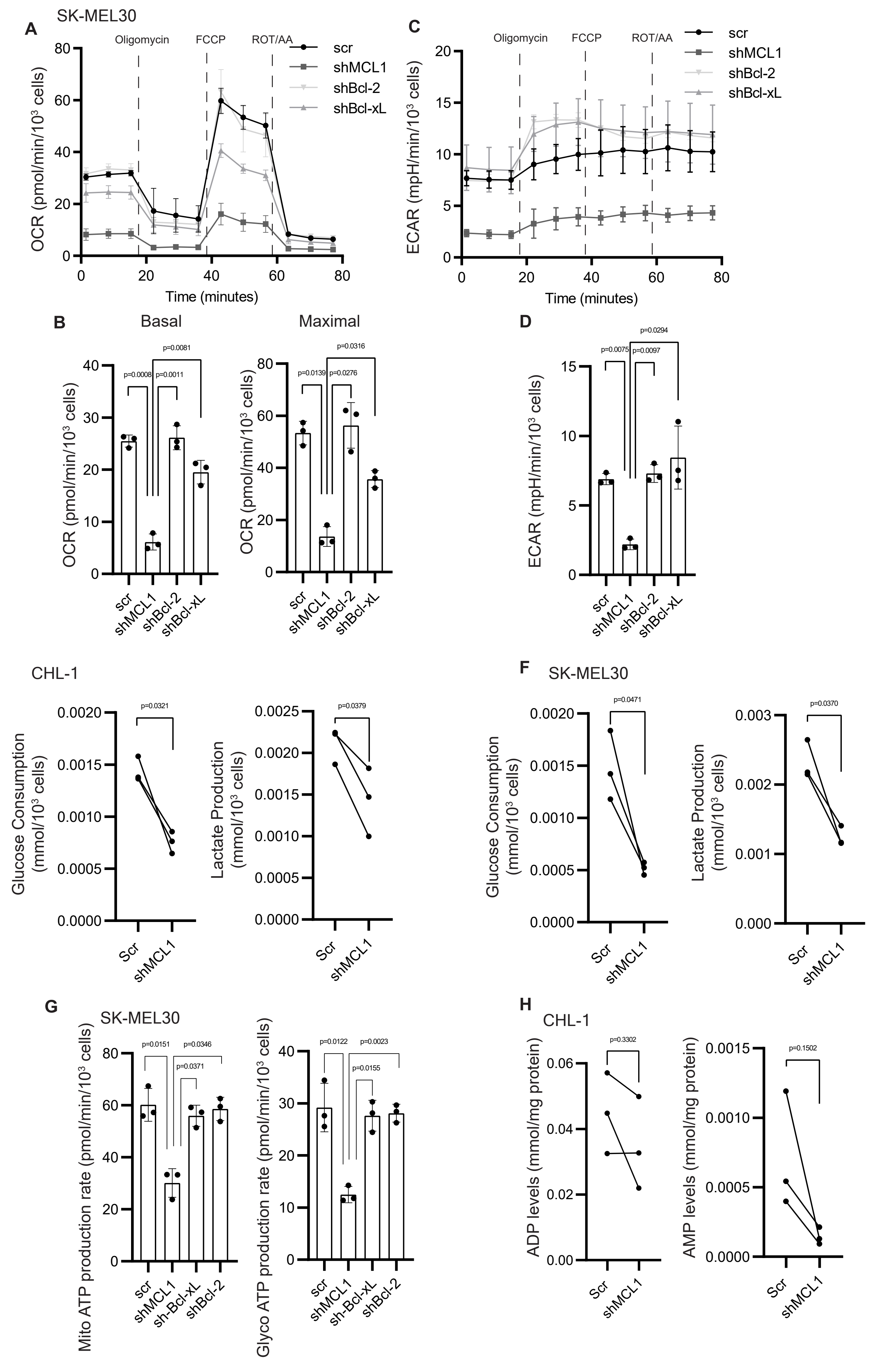
SK-MEL30
A
Oligomycin
FCCP
ROT/AA
scr
shMCL1
shBcl-2
shBcl-xL
OCR (pmol/min/10³ cells)
Time (minutes)
C
ECAR (mpH/min/10³ cells)
B
Basal
Maximal
p=0.0008
p=0.0011
p=0.0081
p=0.0139
p=0.0276
p=0.0316
D
p=0.0075
p=0.0097
p=0.0294
ECAR (mpH/min/10³ cells)
E
CHL-1
p=0.0321
p=0.0379
Glucose Consumption (mmol/10³ cells)
Lactate Production (mmol/10³ cells)
Scr
shMCL1
F
SK-MEL30
p=0.0471
p=0.0370
G
SK-MEL30
p=0.0151
p=0.0346
p=0.0371
p=0.0122
p=0.0023
p=0.0155
Mito ATP production rate (pmol/min/10³ cells)
Glyco ATP production rate (pmol/min/10³ cells)
sh-Bcl-xL
H
CHL-1
p=0.3302
p=0.1502
ADP levels (mmol/mg protein)
AMP levels (mmol/mg protein)

Supplementary Fig. 3. MCL1 controls cellular bioenergetics.

(A, B) Oxygen consumption rate (OCR) (A), basal and maximal mitochondrial respiration (B) measured by Seahorse XF Mito Stress Test of SK-MEL30 cells transduced with the indicated shRNAs for 72 hours. (n = 3 biologically independent samples). **(C, D)** Extracellular acidification rate (ECAR) of SK-MEL30 cells measured as in A. (n = 3 biologically independent samples). **(E, F)** Glucose consumption and lactate production of CHL-1 (E) and SK-MEL30 (F) cells transduced with either scrambled shRNA or shRNA against MCL1 and incubated for 72 hours. Values are normalized to cell numbers. Lines connect values from independent repeats. (n = 3 biologically independent samples). **(G)** Mitochondria- and glycolysis- derived ATP production rate measured by Seahorse XF Real-Time ATP Rate Assay of SK-MEL30 cells transduced with the indicated shRNAs for 72 hours. (n = 3 biologically independent samples). **(H)** ADP and AMP levels measured using LC-MS/MS and normalized to protein concentrations in CHL-1 cells transduced with either scrambled shRNA or shRNA against MCL1. Lines connect values from independent repeats. (n = 3 biologically independent samples). Data is presented as mean +/- SD and significance is determined by (ratio) paired two-tailed t-test.

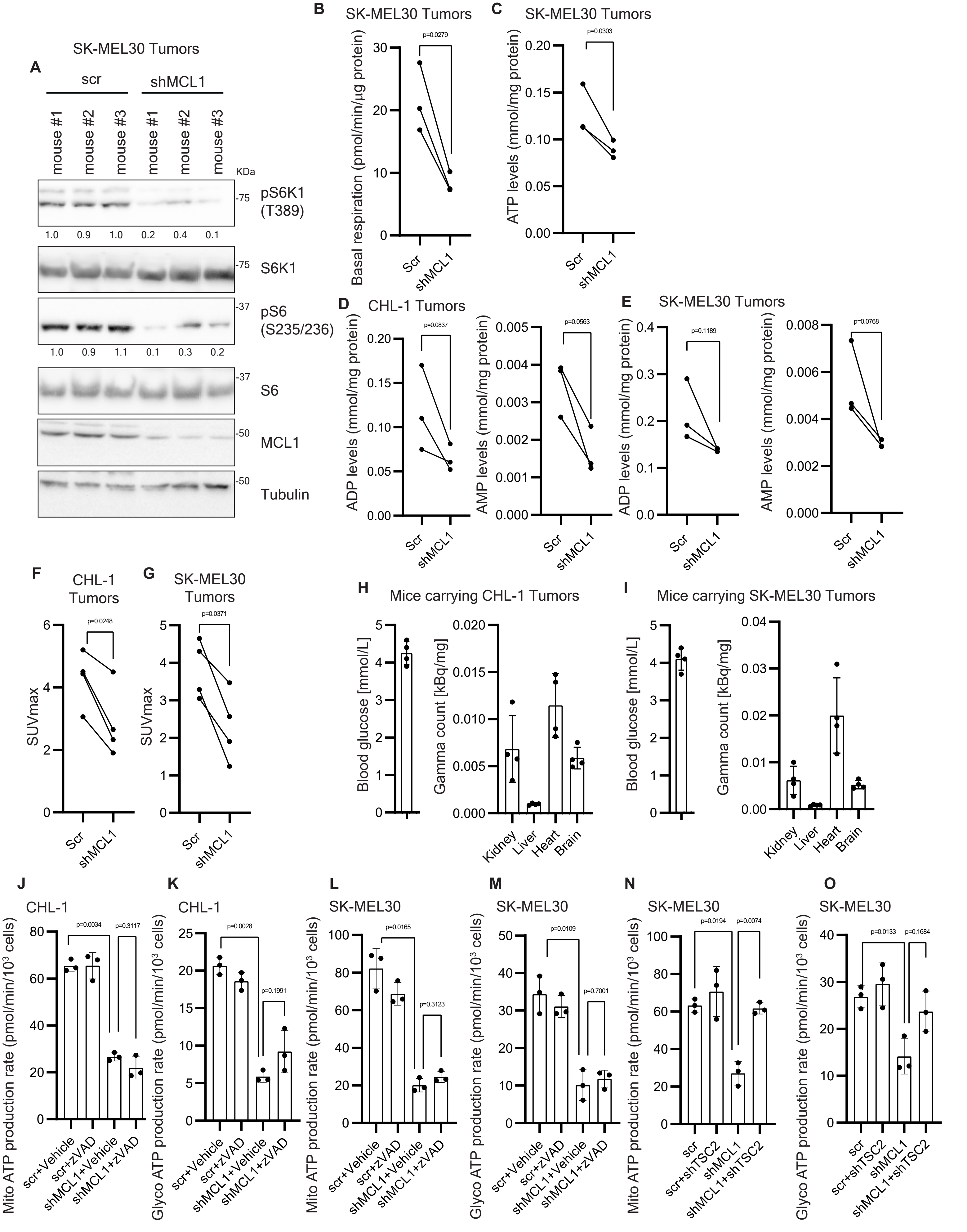

A
SK-MEL30 Tumors
scr
shMCL1
mouse #1
mouse #2
mouse #3
KDa
pS6K1 (T389)
1.0 0.9 1.0 0.2 0.4 0.1
S6K1
pS6 (S235/236)
1.0 0.9 1.1 0.1 0.3 0.2
S6
MCL1
Tubulin
B
SK-MEL30 Tumors
p=0.0279
Basal respiration (pmol/min/µg protein)
Scr
shMCL1
C
SK-MEL30 Tumors
p=0.0303
ATP levels (mmol/mg protein)
D
CHL-1 Tumors
p=0.0837
ADP levels (mmol/mg protein)
p=0.0563
AMP levels (mmol/mg protein)
E
SK-MEL30 Tumors
p=0.1189
p=0.0768
F
CHL-1 Tumors
p=0.0248
SUVmax
G
SK-MEL30 Tumors
p=0.0371
H
Mice carrying CHL-1 Tumors
Blood glucose [mmol/L]
Gamma count [kBq/mg]
Kidney
Liver
Heart
Brain
I
Mice carrying SK-MEL30 Tumors
J
CHL-1
p=0.0034
p=0.3117
Mito ATP production rate (pmol/min/10³ cells)
scr+Vehicle
scr+zVAD
shMCL1+Vehicle
shMCL1+zVAD
K
CHL-1
p=0.0028
p=0.1991
Glyco ATP production rate (pmol/min/10³ cells)
L
SK-MEL30
p=0.0165
p=0.3123
M
SK-MEL30
p=0.0109
p=0.7001
N
SK-MEL30
p=0.0194
p=0.0074
scr
scr+shTSC2
shMCL1
shMCL1+shTSC2
O
SK-MEL30
p=0.0133
p=0.1684

Supplementary Fig. 4. MCL1-mTORC1 axis regulates tumor bioenergetics

(A) Immunoblotting analysis of lysates prepared from subcutaneous xenografts established in both flanks of mice with SK-MEL30 cells transduced with either scrambled shRNA or doxycycline-inducible shRNA against MCL1. After establishment of xenografts, mice were kept on 1 mg/ml doxycycline supplemented in the drinking water for 5 days to induce MCL1 shRNA. The samples derived from the same experiment but different gels for pS6, pS6K1, another for S6, S6K1 and another for MCL1 were processed in parallel. **(B)** Basal mitochondrial respiration normalized to protein concentrations of tumors derived from SK-MEL30 cells as in (A). Tumors were isolated, immediately dissociated using GentleMACS tissue dissociator, plated in poly-D-lysine coated Seahorse plates and measured by Seahorse XF. (n = mice per group). **(C)** ATP levels measured using LC-MS/MS and normalized to protein concentrations in tumors derived from CHL-1 cells as in (A). Lines connect values from the two tumors established on both flanks of the same mouse. (n = 3 mice per group). **(D, E)** ADP and AMP levels measured using LC-MS/MS and normalized to protein concentrations in tumors derived from CHL-1 (D) or SK-MEL30 (E) cells as in (A). Lines connect values from the two tumors established on both flanks of the same mouse. (n = 3 mice prer group). **(F, G)** ^{18}FDG-PET Maximum Standardized Uptake Values (SUVmax) of tumors derived from control (scr) or MCL1-depleted (shMCL1) CHL-1 cells (F) or SK-MEL30 (G) cells. Values were normalized to body weight. (n = 4 mice per group). **(H, I)** Blood glucose levels and qualification of gamma radiation count normalized to administrated ^{18}FDG and organ weight [kBq/mg] of different organs isolated from mice carrying control and MCL1-depleted tumors established as in (A) from CHL-1 (H) or SK-MEL 30 (I) cells followed by 18FDG-PET assay and finally isolation of organs and measurement of gamma radiation using automated gamma counter. Values were normalized for injected ^{18}FDG. (n = 4 mice per group). **(J-M)** Mitochondria- and glycolysis- derived ATP production rate measured by Seahorse XF Real-Time ATP Rate Assay of CHL-1 (J,K) and SK-MEL30 (L,M) cells transduced with either scrambled shRNA or shRNA against MCL1 for 72 hours and treated with or without pan-caspase inhibitor zVAD-fmk (20µM) for the last 16 hours. (n = 3 biologically independent samples). **(N, O)** Mitochondria- (N) and glycolysis- derived (O) ATP production rate measured by Seahorse XF Real-Time ATP Rate Assay of SK-MEL30 cells transduced as indicated. (n = 3 biologically independent samples). Data is presented as mean +/- SD and significance is determined by (ratio) paired two-tailed t-test.

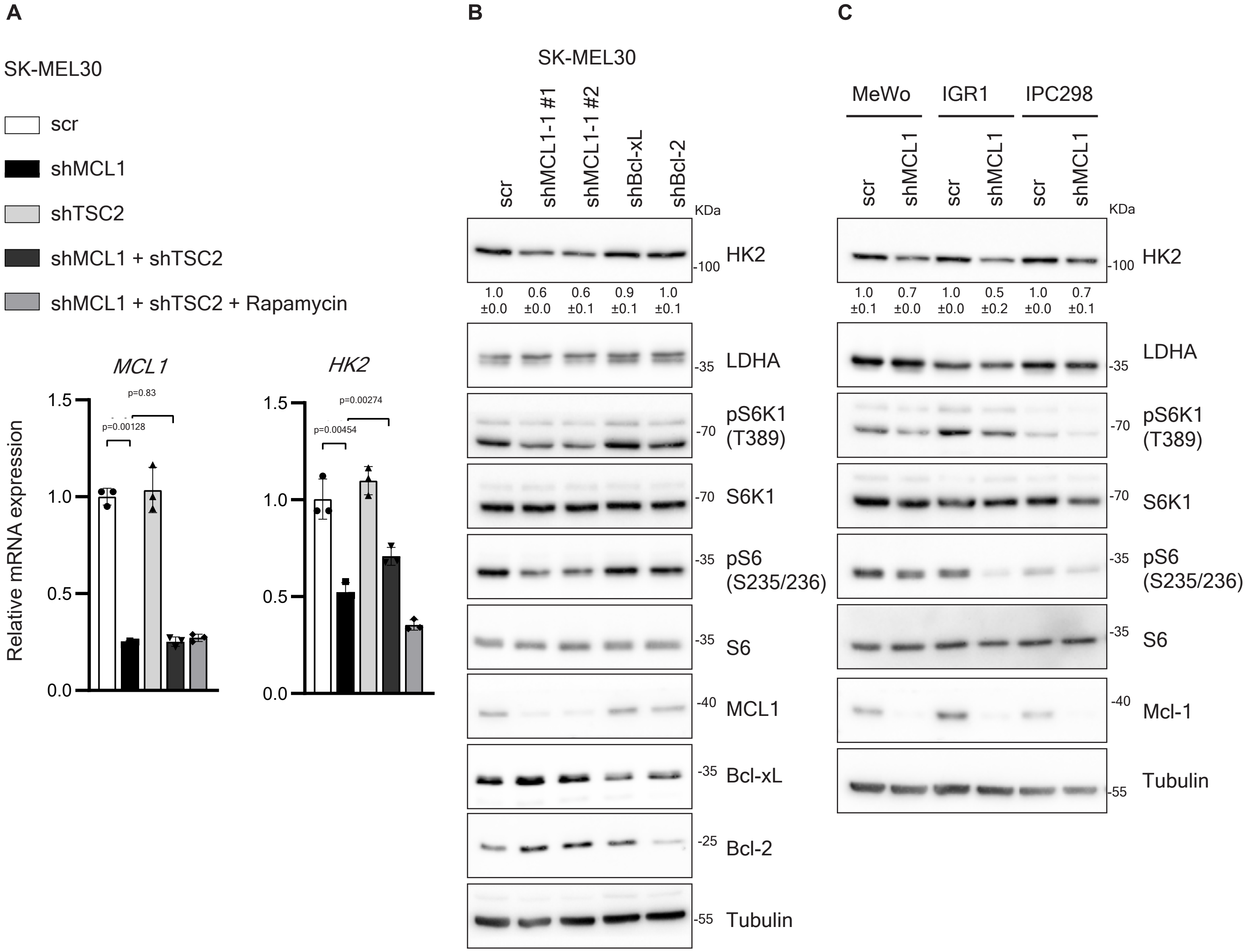
A
SK-MEL30
scr
shMCL1
shTSC2
shMCL1 + shTSC2
shMCL1 + shTSC2 + Rapamycin
MCL1
HK2
Relative mRNA expression
1.5
1.0
0.5
0.0
p=0.83
p=0.00128
p=0.00274
p=0.00454
B
SK-MEL30
scr
shMCL1-1 #1
shMCL1-1 #2
shBcl-xL
shBcl-2
KDa
HK2
-100
1.0 ±0.0
0.6 ±0.0
0.6 ±0.1
0.9 ±0.1
1.0 ±0.1
LDHA
-35
pS6K1 (T389)
-70
S6K1
-70
pS6 (S235/236)
-35
S6
-35
MCL1
-40
Bcl-xL
-35
Bcl-2
-25
Tubulin
-55
C
MeWo
IGR1
IPC298
scr
shMCL1
KDa
HK2
-100
1.0 ±0.1
0.7 ±0.0
1.0 ±0.0
0.5 ±0.2
1.0 ±0.0
0.7 ±0.1
LDHA
-35
pS6K1 (T389)
-70
S6K1
-70
pS6 (S235/236)
-35
S6
-35
Mcl-1
-40
Tubulin
-55

Supplementary Fig. 5. MCL1-mTORC1 axis regulates HK2.

(A) Relative mRNA expression levels (normalized to beta-actin) of MCL1 and HK2 assessed by real-time qPCR in SK-MEL30 cells transduced with the indicated shRNAs and treated with or without Rapamycin (50 nM) for 24 hours. (n = 3 biologically independent samples). Data is presented as mean +/- SEM and significance is determined by paired two-tail t-test. **(B, C)** Immunoblotting analysis of lysate derived from SK-MEL30 (B), MeWo, IGR1 or IPC298 (C) melanoma cells transduced with the indicated shRNAs for 72 hours. The samples derived from the same experiment but different gels for HK2, pS6K1, MCL1, Bcl-XL, BCL2, another for S6K1, pS6, LDHA and another for S6 were processed in parallel (B) The samples derived from the same experiment but different gels for HK2, pS6K1, MCL1, another for S6K1, pS6 and another for S6, LDHA were processed in parallel (C).

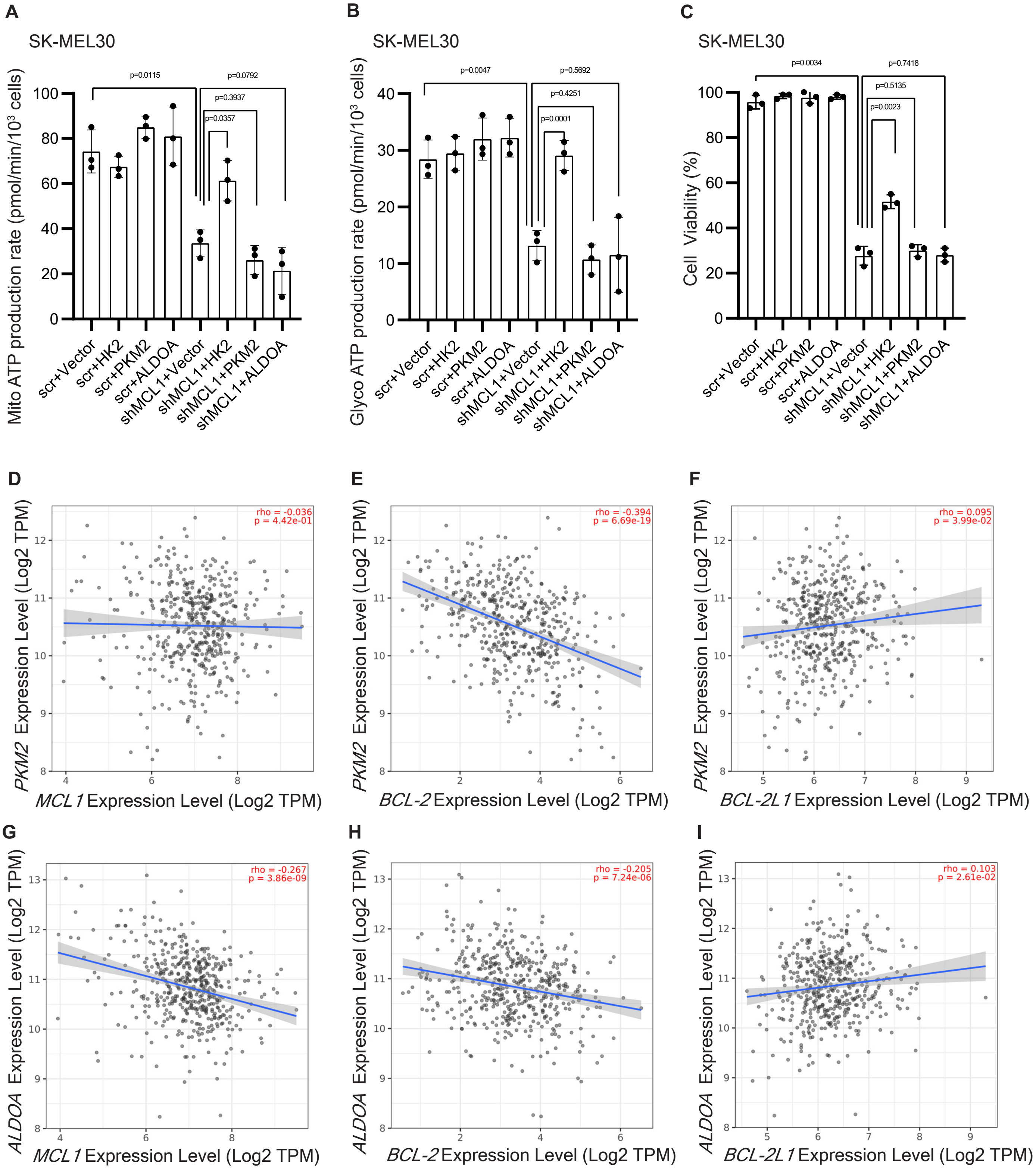
A
SK-MEL30
Mito ATP production rate (pmol/min/10³ cells)
p=0.0115
p=0.0792
p=0.3937
p=0.0357
scr+Vector
scr+HK2
scr+PKM2
scr+ALDOA
shMCL1+Vector
shMCL1+HK2
shMCL1+PKM2
shMCL1+ALDOA
B
SK-MEL30
Glyco ATP production rate (pmol/min/10³ cells)
p=0.0047
p=0.5692
p=0.4251
p=0.0001
C
SK-MEL30
Cell Viability (%)
p=0.0034
p=0.7418
p=0.5135
p=0.0023
D
rho = -0.036
p = 4.42e-01
PKM2 Expression Level (Log2 TPM)
MCL1 Expression Level (Log2 TPM)
E
rho = -0.394
p = 6.69e-19
PKM2 Expression Level (Log2 TPM)
BCL-2 Expression Level (Log2 TPM)
F
rho = 0.095
p = 3.99e-02
PKM2 Expression Level (Log2 TPM)
BCL-2L1 Expression Level (Log2 TPM)
G
rho = -0.267
p = 3.86e-09
ALDOA Expression Level (Log2 TPM)
MCL1 Expression Level (Log2 TPM)
H
rho = -0.205
p = 7.24e-06
ALDOA Expression Level (Log2 TPM)
BCL-2 Expression Level (Log2 TPM)
I
rho = 0.103
p = 2.61e-02
ALDOA Expression Level (Log2 TPM)
BCL-2L1 Expression Level (Log2 TPM)

Supplementary Fig. 6. MCL1-mTORC1 axis regulates metabolism via HK2.

(A,B) Mitochondria- (A) and glycolysis- derived (B) ATP production rate measured by Seahorse XF Real-Time ATP Rate Assay of SK-MEL30 cells overexpressing glycolysis regulators HK2, PKM2, ALDOA or vector and transduced with either scrambled shRNA or shRNA against MCL1 for 72 hours. (n = 3 biologically independent samples). **(C)** Percentage of cell viability of SK-MEL30 cells transduced as in (A) after 96 hours in culture. (n = 3 biologically independent samples). Data presented as mean +/- SD and significance is determined by paired two-tailed t-test. **(D-F)** Correlation between the mRNA levels of PKM2 and MCL1 (D), BCL-2 (E) and BCL-2L1 (F) in The Cancer Genome Atlas (TCGA TCGA-SKCM (n = 471 patients) analyzed using TIMER2.0 (http://timer.cistrome.org/). Spearman's rho value was used to evaluate the degree of their correlation. **(G-I)** Correlation between the mRNA levels of ALDOA and MCL1 (G), BCL-2 (H) and BCL-2L1 (I) in The Cancer Genome Atlas (TCGA TCGA-SKCM (n = 471 patients) analyzed using TIMER2.0 (http://timer.cistrome.org/). Spearman's rho value was used to evaluate the degree of their correlation.

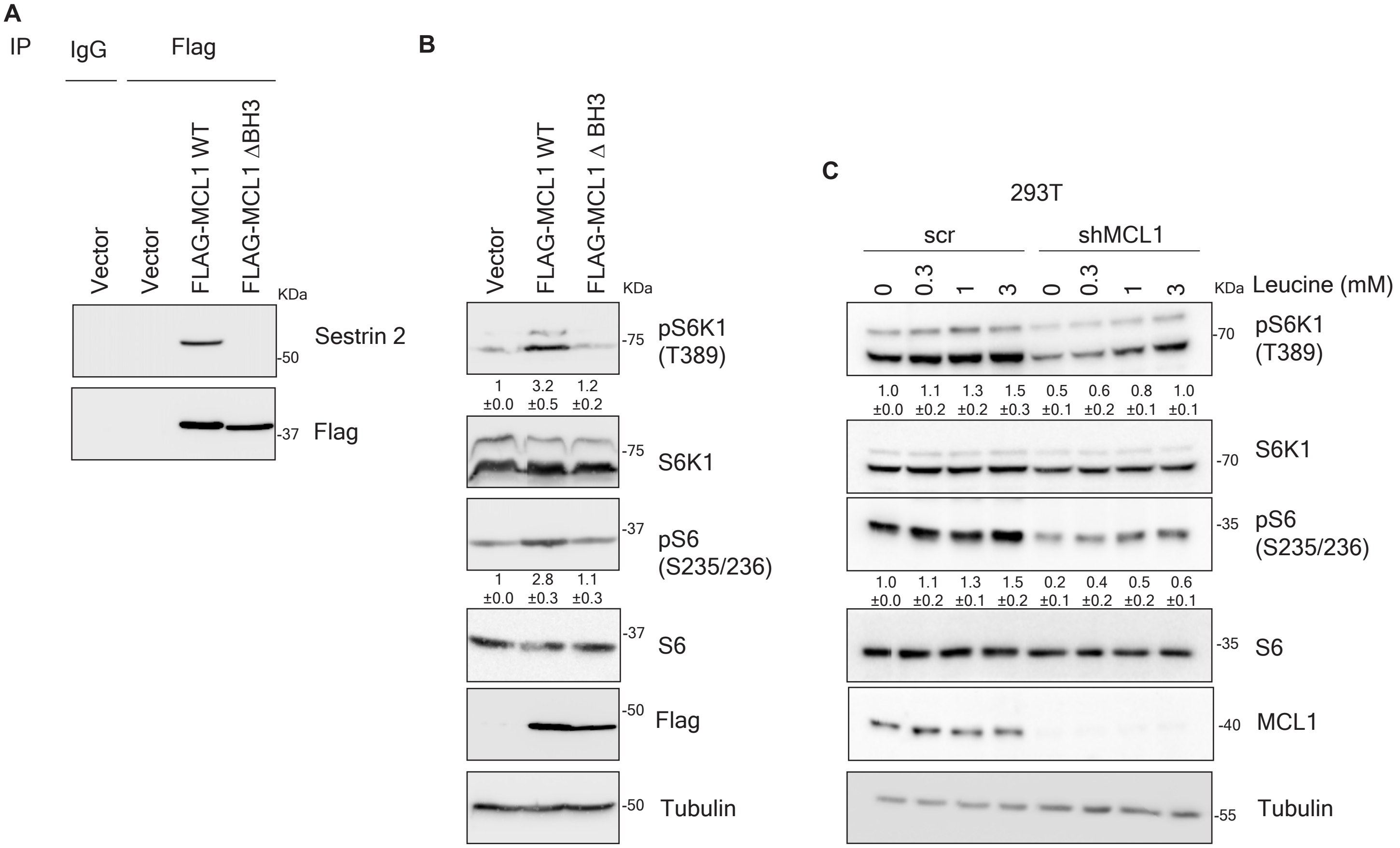
A
IP
IgG
Flag
Vector
Vector
FLAG-MCL1 WT
FLAG-MCL1 ΔBH3
KDa
Sestrin 2
-50
-37
Flag
B
Vector
FLAG-MCL1 WT
FLAG-MCL1 Δ BH3
KDa
-75
pS6K1 (T389)
1 ±0.0
3.2 ±0.5
1.2 ±0.2
-75
S6K1
-37
pS6 (S235/236)
1 ±0.0
2.8 ±0.3
1.1 ±0.3
-37
S6
-50
Flag
-50
Tubulin
C
293T
scr
shMCL1
0
0.3
1
3
0
0.3
1
3
KDa
Leucine (mM)
-70
pS6K1 (T389)
1.0 ±0.0
1.1 ±0.2
1.3 ±0.2
1.5 ±0.3
0.5 ±0.1
0.6 ±0.2
0.8 ±0.1
1.0 ±0.1
-70
S6K1
-35
pS6 (S235/236)
1.0 ±0.0
1.1 ±0.2
1.3 ±0.1
1.5 ±0.2
0.2 ±0.1
0.4 ±0.2
0.5 ±0.2
0.6 ±0.1
-35
S6
-40
MCL1
-55
Tubulin

Supplementary Fig. 7. MCL1 binds Sestrin 2.

(A) Immunoprecipitation of Flag-tagged wild-type MCL1 or a Flag-tagged construct of MCL1 lacking the . BH3 binding pocket from 293T cells using anti-Flag resin or IgG as control. **(B)** Immunoblotting analysis of total cell lysates used in (A). The samples derived from the same experiment but different gels for pS6, pS6K1, another for S6, S6K1 and another for FLAG were processed in parallel. **(C)** Immunoblotting of 293T cells transduced with scrambled shRNA or shRNA against MCL1 and treated with the indicated concentrations of Leucine for 1.5 hours before lysing the cells. The samples derived from the same experiment but different gels for pS6K1, MCL1, pS6, and another for S6K1, S6 were processed in parallel.

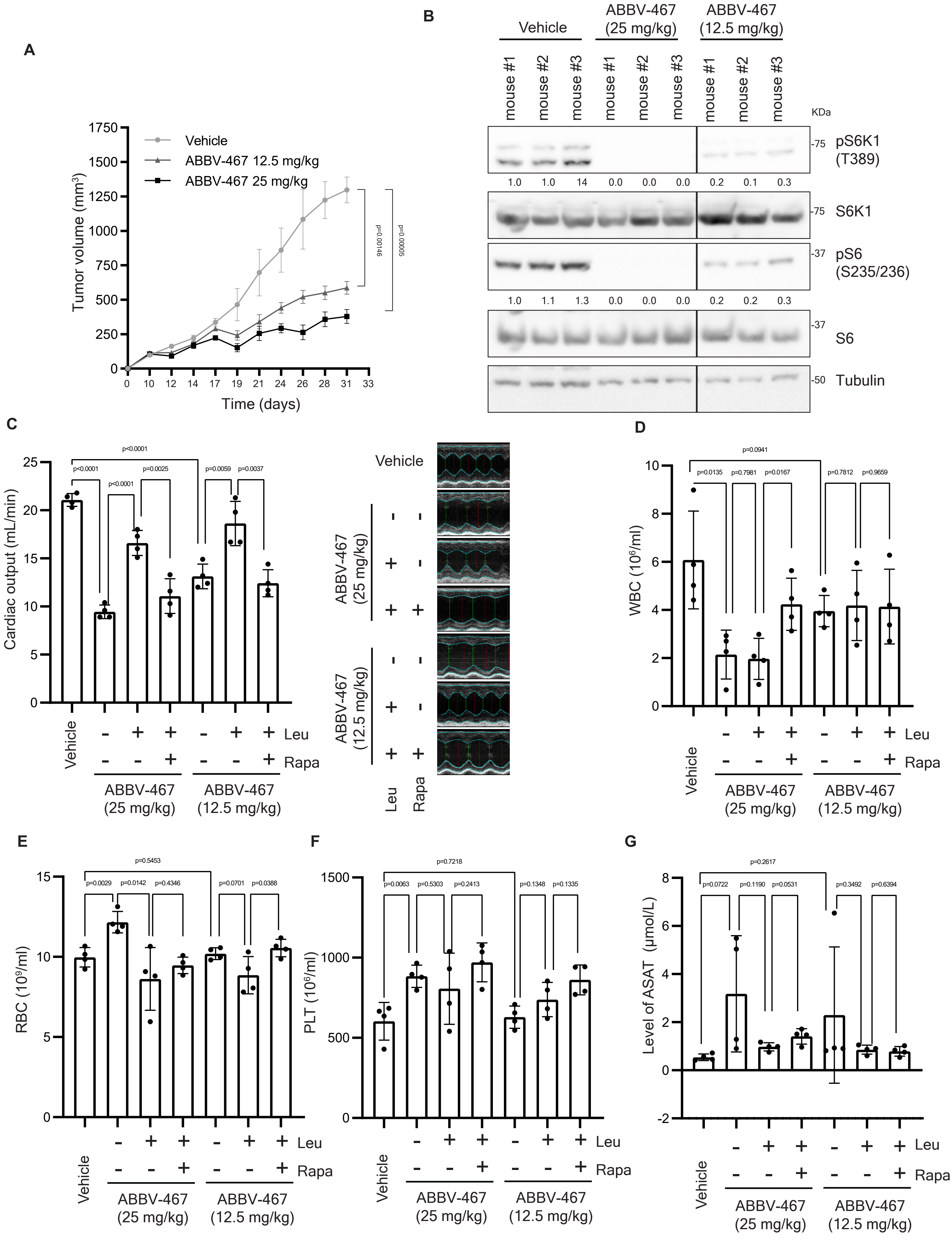

A
B
C
D
E
F
G
Vehicle
ABBV-467 12.5 mg/kg
ABBV-467 25 mg/kg
Tumor volume (mm³)
Time (days)
p=0.00146
p=0.00005
ABBV-467 (25 mg/kg)
ABBV-467 (12.5 mg/kg)
mouse #1
mouse #2
mouse #3
KDa
pS6K1 (T389)
S6K1
pS6 (S235/236)
S6
Tubulin
-75
-37
-50
1.0 1.0 14 0.0 0.0 0.0 0.2 0.1 0.3
1.0 1.1 1.3 0.0 0.0 0.0 0.2 0.2 0.3
Cardiac output (mL/min)
p<0.0001
p=0.0025
p=0.0059
p=0.0037
Leu
Rapa
WBC (10⁶/ml)
p=0.0941
p=0.0135
p=0.7981
p=0.0167
p=0.7812
p=0.9659
RBC (10⁹/ml)
p=0.5453
p=0.0029
p=0.0142
p=0.4346
p=0.0701
p=0.0388
PLT (10⁶/ml)
p=0.7218
p=0.0063
p=0.5303
p=0.2413
p=0.1348
p=0.1335
Level of ASAT (µmol/L)
p=0.2617
p=0.0722
p=0.1190
p=0.0531
p=0.3492
p=0.6394

Supplementary Fig. 8. Inhibition of mTORC1 contributes to the cardiotoxicity induced by MCL1 inhibitor ABBV-467.

(A) Growth rate of subcutaneous xenografts established in NSG mice from CHL-1 cells. After establishment of xenografts, mice were treated with Vehicle or ABBV-467 (25 mg/kg or 12.5 mg/kg administrated by I.V. injection on a Q7D × 3 schedule. (n = 3 mice per group). **(B)** Immunoblotting analysis of lysates derived from tumors in (A). Bands are all from the same blots with the same exposure time. The samples derived from the same experiment but different gels for pS6, pS6K1 and another for S6, S6K1 were processed in parallel. **(C)** Cardiac output and representative echocardiograph images of Humanized Mcl-1 mice treated for three weeks with Vehicle or ABBV-467 (25mg/kg or 12.5 mg/kg administrated by I.V. injection on a Q7D × 3 schedule) alone or in the indicated combinations with supplementation of Leucine (150 mmol/L in the drinking water) and treatment with Rapamycin (2 mg/kg I.P. three times a week). (n = 4 mice per group). **(D-G)** Count of White Blood Cells (WBC) (D), Red Blood Cells (RBC) (E), Platelets (PLT) (F) and serum levels of ASAT (G) of mice in (C). (n = 4 mice per group). Data is presented as mean +/- SD and significance is determined by unpaired two-tailed t-test.

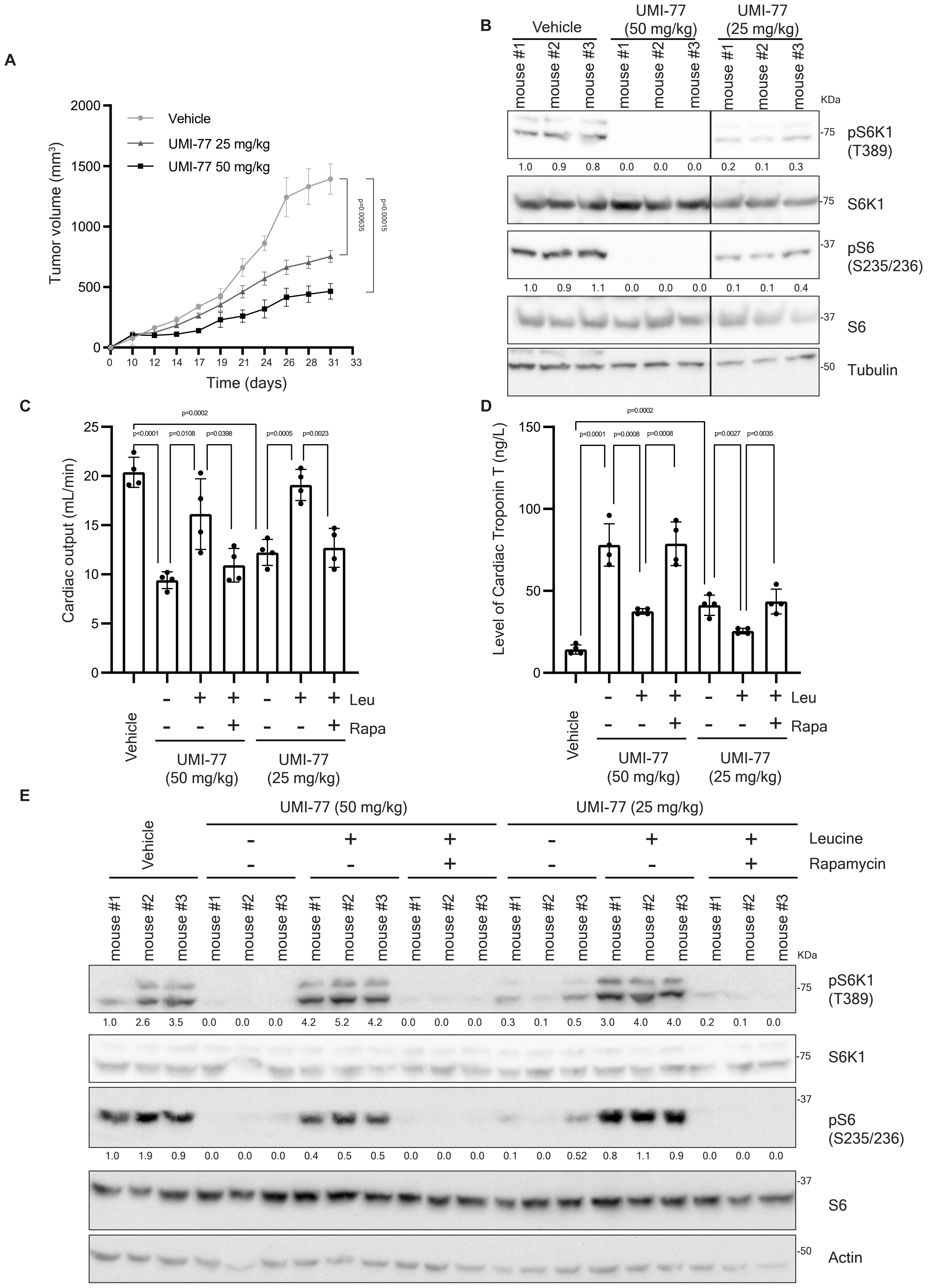

A
B
C
D
E
Tumor volume (mm³)
Time (days)
Vehicle
UMI-77 25 mg/kg
UMI-77 50 mg/kg
p=0.00635
p=0.0015
Vehicle
UMI-77 (50 mg/kg)
UMI-77 (25 mg/kg)
mouse #1
mouse #2
mouse #3
KDa
pS6K1 (T389)
S6K1
pS6 (S235/236)
S6
Tubulin
1.0 0.9 0.8 0.0 0.0 0.0 0.2 0.1 0.3
1.0 0.9 1.1 0.0 0.0 0.0 0.1 0.1 0.4
Cardiac output (mL/min)
p=0.0002
p<0.0001
p=0.0108
p=0.0398
p=0.0005
p=0.0023
Leu
Rapa
Level of Cardiac Troponin T (ng/L)
p=0.0002
p<0.0001
p=0.0008
p=0.0008
p=0.0027
p=0.0035
Leucine
Rapamycin
Actin
1.0 2.6 3.5 0.0 0.0 0.0 4.2 5.2 4.2 0.0 0.0 0.0 0.3 0.1 0.5 3.0 4.0 4.0 0.2 0.1 0.0
1.0 1.9 0.9 0.0 0.0 0.0 0.4 0.5 0.5 0.0 0.0 0.0 0.1 0.0 0.52 0.8 1.1 0.9 0.0 0.0 0.0

Supplementary Fig. 9. Inhibition of mTORC1 contributes to the cardiotoxicity induced by MCL1 inhibitor UMI-77.

(A) Growth rate of subcutaneous xenografts established in NSG mice from CHL-1 cells. After establishment of xenografts, mice were treated with Vehicle or UMI-77 (50 mg/kg or 25 mg/kg administrated by I.P. injection three times a week. (n = 3 mice per group). **(B)** Immunoblotting analysis of lysates derived from tumors in (A). Bands are all from the same blots with the same exposure time. The samples derived from the same experiment but different gels for pS6, pS6K1 and another for S6, S6K1 were processed in parallel. **(C, D)** Cardiac output (C) and serum levels of cardiac-specific Troponin T (D), of C57BL/6 mice treated with Vehicle or UMI-77 (50 mg/kg or 25 mg/kg administrated by I.P. injection 3 times a week for 4 weeks) alone or in the indicated combinations with supplementation of Leucine (150 mmol/L in the drinking water) and treatment with Rapamycin (2 mg/kg I.P. three times a week). (n = 4 mice per group). **(E)** Immunoblotting of lysate derived from the hearts of mice in A. The samples derived from the same experiment but different gels for pS6, pS6K1, another for S6 and another for S6K1 were processed in parallel. Data presented as mean +/- SD and significance is determined by unpaired two-tailed t-test.

		EF (%)	FS (%)	LVIDs (mm)	LVIDd (mm)	LVESV (ul)	LVEDV (ul)	LV Mass (mg)	LV Mass cor (mg)
**Vehicle**	**Mean**	64.475	35.475	2.55	3.9	24.75	67.45	124.525	99.65
	**SD**	12.191	8.934	0.662	0.551	15.059	21.721	9.925	7.933
**ABBV-467 (25mg/kg)**	**Mean**	54.075	27.475	2.4175	3.3075	21.6	45.1	82.15	65.725
	**SD**	11.193	7.078	0.485	0.380	10.385	12.581	12.307	9.865
**ABBV-467 (25mg/kg)+Leu**	**Mean**	79.775	47.2	1.6325	3.0875	7.67	37.6825	116.01	92.825
	**SD**	4.022	4.068	0.163	0.148	1.953	4.299	31.665	25.345
**ABBV-467 (25mg/kg)+Leu+Rapa**	**Mean**	51.175	25.625	2.7825	3.735	29.9425	60.51	110.625	88.325
	**SD**	5.321	3.161	0.420	0.467	11.228	18.642	7.881	6.127
**ABBV-467 (12.5mg/kg)**	**Mean**	54.975	28.225	2.6575	3.695	26	57.975	133.225	106.575
	**SD**	3.398	2.341	0.109	0.122	2.601	4.534	26.451	21.209
**ABBV-467 (12.5mg/kg)+Leu**	**Mean**	61.925	32.825	2.54	3.765	23.725	61.35	120.575	73.325
	**SD**	5.713	3.930	0.319	0.274	7.690	11.216	20.374	36.510
**ABBV-467 (12.5mg/kg)+Leucine+Rapa**	**Mean**	60.55	31.675	2.4225	3.54	20.7	52.425	109.775	87.75
	**SD**	4.812	3.342	0.148	0.114	3.209	3.984	11.501	9.340

Supplementary Table 1. Echocardiography parameters of humanized Mcl-1 mice treated with MCL1 inhibitor ABBV-467.

Humanized Mcl-1 mice were treated for three weeks with Vehicle or ABBV-467 (25mg/kg or 12.5 mg/kg administrated by I.V. injection on a Q7D × 3 schedule) alone or in the indicated combinations with supplementation of Leucine (150 mmol/L in the drinking water) and treatment with Rapamycin (2 mg/kg I.P. three times a week). (n = 4 mice per group). Data is presented as mean +/- SD. EF: Ejection Fraction; FS: Fraction Shortening; LVID: Left Ventricular Internal Dimension; LVESV: Left Ventricular End-Systole Volume; LVEDV: Left Ventricular End-Diastolic Volume; LV mass: Left Ventricular Mass; LV mass cor. : Left Ventricular Mass corrected (for body size).

		ABBV-467 (25 mg/kg)			ABBV-467 (12.5 mg/kg)			
	Vehicle	-	+	+	-	+	+	Leu
		-	-	+	-	-	+	Rapa
Mouse 1	<15	<15	36	<15	<15	21	<15	
Mouse 2	<15	<15	27	18	<15	<15	<15	
Mouse 3	<15	<15	<15	<15	24	<15	<15	
Mouse 4	<15	<15	<15	39	<15	27	<15	

Supplementary Table 2. Creatinine levels in humanized Mcl-1 mice treated with MCL1 inhibitor ABBV-467.

Humanized Mcl-1 mice were treated for three weeks with Vehicle or ABBV-467 (25mg/kg or 12.5 mg/kg administrated by I.V. injection on a Q7D × 3 schedule) alone or in the indicated combinations with supplementation of Leucine (150 mmol/L in the drinking water) and treatment with Rapamycin (2 mg/kg I.P. three times a week). (n = 4 mice per group).

		EF (%)	FS (%)	LVIDs (mm)	LVIDd (mm)	LVESV (ul)	LVEDV (ul)	LV Mass (mg)	LV Mass cor (mg)
**Vehicle**	**Mean**	74.8	43.333	2	3.5	13.633	51.2	129.67	103.7
	**SD**	10.068	8.461	0.489	0.326	8.240	11.676	8.829	7.036
**UMI-77 (50mg/kg)**	**Mean**	74.433	42.1	1.74	2.99	9.133	34.967	82.667	66.133
	**SD**	5.358	4.618	0.252	0.205	3.308	5.801	9.218	7.414
**UMI-77 (50mg/kg)+Leu**	**Mean**	66.8	36.733	2.32	3.667	19.033	57.267	120.833	96.7
	**SD**	10.671	8.100	0.341	0.285	6.429	11.019	7.220	5.745
**UMI-77 (50mg/kg)+Leu+Rapa**	**Mean**	68.667	38.167	2.02	3.23	14.467	42.567	80.033	64
	**SD**	13.255	10.362	0.581	0.397	9.898	12.653	24.707	19.741
**UMI-77 (25mg/kg)**	**Mean**	67.767	36.633	2.037	3.21	13.4	41.4	88.9	71.133
	**SD**	1.464	1.124	0.101	0.128	1.646	3.863	12.630	10.129
**UMI-77 (25mg/kg)+Leu**	**Mean**	72.633	40.967	2.087	3.527	14.433	51.967	110.4	88.3
	**SD**	5.331	4.366	0.260	0.182	4.509	6.451	8.316	6.664
**UMI-77 (25mg/kg)+Leu+Rapa**	**Mean**	67	46.9	2.073	4.947	6.36	41.167	59.767	54.45
	**SD**	22.050	8.485	0.894	3.436	4.541	13.742	19.094	14.213

Supplementary Table 3. Echocardiography parameters of C57BL/6 mice treated with MCL1 inhibitor UMI-77.

C57BL/6 mice treated with Vehicle or UMI-77 (50 mg/kg or 25 mg/kg administrated by I.P. injection 3 times a week for 4 weeks) alone or in the indicated combinations with supplementation of Leucine (150 mmol/L in the drinking water) and treatment with Rapamycin (2 mg/kg I.P. three times a week). (n = 4 mice per group). Data is presented as mean +/- SD. EF: Ejection Fraction; FS: Fraction Shortening; LVID: Left Ventricular Internal Dimension; LVESV: Left Ventricular End-Systole Volume; LVEDV: Left Ventricular End-Diastolic Volume; LV mass: Left Ventricular Mass; LV mass cor. : Left Ventricular Mass corrected (for body size).
