# Supplementary material for: MCL1 modulates mTORC1 signaling to promote bioenergetics and tumorigenesis
Source: Nat Commun. 2025 Dec 1;16:10841. doi: 10.1038/s41467-025-66831-4 (PMC12673096; doi:10.1038/s41467-025-66831-4)
Supplement: Supplementary file 2 — Reporting Summary [file 41467_2025_66831_MOESM2_ESM.pdf]

nature portfolio

Corresponding author(s): Mohamed Elgendy

Last updated by author(s): Sep 5, 2025

Reporting Summary

Nature Portfolio wishes to improve the reproducibility of the work that we publish. This form provides structure for consistency and transparency in reporting. For further information on Nature Portfolio policies, see our Editorial Policies and the Editorial Policy Checklist.

Statistics

For all statistical analyses, confirm that the following items are present in the figure legend, table legend, main text, or Methods section.

n/a	Confirmed	
☐	☒	The exact sample size (*n*) for each experimental group/condition, given as a discrete number and unit of measurement
☐	☒	A statement on whether measurements were taken from distinct samples or whether the same sample was measured repeatedly
☐	☒	The statistical test(s) used AND whether they are one- or two-sided<br>*Only common tests should be described solely by name; describe more complex techniques in the Methods section.*
☒	☐	A description of all covariates tested
☒	☐	A description of any assumptions or corrections, such as tests of normality and adjustment for multiple comparisons
☐	☒	A full description of the statistical parameters including central tendency (e.g. means) or other basic estimates (e.g. regression coefficient) AND variation (e.g. standard deviation) or associated estimates of uncertainty (e.g. confidence intervals)
☐	☒	For null hypothesis testing, the test statistic (e.g. F, t, r) with confidence intervals, effect sizes, degrees of freedom and P value noted<br>*Give P values as exact values whenever suitable.*
☒	☐	For Bayesian analysis, information on the choice of priors and Markov chain Monte Carlo settings
☒	☐	For hierarchical and complex designs, identification of the appropriate level for tests and full reporting of outcomes
☒	☐	Estimates of effect sizes (e.g. Cohen's *d*, Pearson's *r*), indicating how they were calculated

Our web collection on statistics for biologists contains articles on many of the points above.

Software and code

Policy information about availability of computer code

Data collection	No software was used
Data analysis	GraphPad Prism 7 was used for statistical analysis and data plotting

For manuscripts utilizing custom algorithms or software that are central to the research but not yet described in published literature, software must be made available to editors and reviewers. We strongly encourage code deposition in a community repository (e.g. GitHub). See the Nature Portfolio guidelines for submitting code & software for further information.

Data

Policy information about availability of data

All manuscripts must include a data availability statement. This statement should provide the following information, where applicable:

- Accession codes, unique identifiers, or web links for publicly available datasets
- A description of any restrictions on data availability
- For clinical datasets or third party data, please ensure that the statement adheres to our policy

Data Availability Statement is included. All data supporting the findings of this study are available within the manuscript and its Supplementary data. Sequences of primers and shRNAs are described in the Methods Section.

Research involving human participants, their data, or biological material

Policy information about studies with human participants or human data. See also policy information about sex, gender (identity/presentation), and sexual orientation and race, ethnicity and racism.

Reporting on sex and gender	n/a
Reporting on race, ethnicity, or other socially relevant groupings	n/a
Population characteristics	n/a
Recruitment	n/a
Ethics oversight	*Identify the organization(s) that approved the study protocol.*

Note that full information on the approval of the study protocol must also be provided in the manuscript.

Field-specific reporting

Please select the one below that is the best fit for your research. If you are not sure, read the appropriate sections before making your selection.

☒ Life sciences ☐ Behavioural & social sciences ☐ Ecological, evolutionary & environmental sciences

For a reference copy of the document with all sections, see nature.com/documents/nr-reporting-summary-flat.pdf

Life sciences study design

All studies must disclose on these points even when the disclosure is negative.

Sample size	The sample size varies among experiments but for most experiments 4-5 mice were used in each group. Specifics are provided in the figures. The size was selected based on prior experience, practical considerations and commitment to the principles of 3Rs.
Data exclusions	No data were excluded from analysis.
Replication	Experiments were repeated at least 3 times, often more. Results were consistently found reproducible.
Randomization	Mice were randomly assigned to different groups.
Blinding	Investigators who performed the measurements of different enzymes in the sera isolated from mice treated with MCL1 inhibitors were blinded to group allocation.

Reporting for specific materials, systems and methods

We require information from authors about some types of materials, experimental systems and methods used in many studies. Here, indicate whether each material, system or method listed is relevant to your study. If you are not sure if a list item applies to your research, read the appropriate section before selecting a response.

Materials & experimental systems

n/a	Involved in the study	
☐	☒	Antibodies
☐	☒	Eukaryotic cell lines
☒	☐	Palaeontology and archaeology
☐	☒	Animals and other organisms
☒	☐	Clinical data
☒	☐	Dual use research of concern
☒	☐	Plants

Methods

n/a	Involved in the study	
☒	☐	ChIP-seq
☒	☐	Flow cytometry
☒	☐	MRI-based neuroimaging

Antibodies

Antibodies used	The following antibodies were used for immunoblotting: anti-Mcl1 582 (Santa Cruz, sc-819, 1:1,000), anti-Bcl2 (Santa Cruz, sc-7382, 1:500), anti-Bcl-xL (CST, catalog 583 no. 2764, 1:1,000), anti-total p70S6kinase (CST, catalog no. 2708, 1:1,000), anti-phospho-584 p70S6kinase (Thr389) (CST, catalog no. 9234, 1:1,000), anti-4E-BP1 (CST, catalog no. 9644, 585 1:10,000), anti-phospho-4E-BP1

	(Thr37/46) (CST, catalog no. 9459, 1:10,000), anti-S6 586 Ribosomal Protein (CST, catalog no. 2217, 1:1,000), anti-phospho-S6 Ribosomal Protein 587 (Ser235/236) (CST, catalog no. 2211, 1:1,000), anti-Hexokinase II (CST, catalog no. 2867, 588 1:1,000), anti-Tubulin (Santa Cruz, sc-271314, 1:1,000), anti-β-Actin (CST, catalog no. 4970, 589 1:2000) and anti-Sestrin 2 (Proteintech, 10795-1-AP, 1:1,000).
Validation	All antibodies used were validated for the used application by the manufactures according to the product data sheets. Additionally, for antibodies used in immunohistochemical analysis, negative controls were obtained by replacing the primary antibody with a non-immune serum at the same concentration.

Eukaryotic cell lines

Policy information about cell lines and Sex and Gender in Research

Cell line source(s)	Internal stock.
Authentication	STR profiling
Mycoplasma contamination	All cell lines were frequently tested for mycoplasma contamination and they all came negative.
Commonly misidentified lines (See ICLAC register)	none.

Animals and other research organisms

Policy information about studies involving animals; ARRIVE guidelines recommended for reporting animal research, and Sex and Gender in Research

Laboratory animals	NOD scid gamma (NSG) , Humanized Mcl-1 and C57BL/6 mice were used in this study.
Wild animals	The study did not involve wild animals.
Reporting on sex	The findings do not apply to one sex.
Field-collected samples	The study did not involve samples collected from the field
Ethics oversight	The experiments were performed in accordance with national and international guidelines for laboratory animal care, approved by the Laboratory Animal Care and Use Committee of the First Faculty of Medicine, Charles University in Prague, and the Ministry of Education, Youth and Sports and of the Czech Republic (MSMT-46307/2020-3) as well as the local ethics committee of Institute of Molecular Genetics of the Czech Academy of Sciences (reference number 39-2022-P).

Note that full information on the approval of the study protocol must also be provided in the manuscript.

Plants

Seed stocks	n/a
Novel plant genotypes	n/a
Authentication	n/a
